# Effect of Different Polishing Systems on the Polishability and Microhardness of an Alkasite Restorative Material

**DOI:** 10.7759/cureus.91719

**Published:** 2025-09-06

**Authors:** Rahaf A Alolayan, Souad A Alfouzan, Asma Munir Khan

**Affiliations:** 1 Pediatric Dentistry, Private Practice, Al Qassim, SAU; 2 Dentistry, Private Practice, Al Qassim, SAU; 3 Operative Dentistry, Qassim University, Buraydah, SAU

**Keywords:** alkasite, bioactive materials, cention n, dental materials, microhardness, polishing systems, restorative dentistry, surface roughness

## Abstract

Objective: In restorative dentistry, the finishing and polishing of restorative materials are important steps to enhance both the aesthetics and longevity of restorations. The aim of this study was to evaluate the effect of different polishing systems on surface roughness and microhardness of the Cention N alkasite restorative material (Ivoclar Vivadent, Schaan, Liechtenstein) and compare its light-cured versus self-cured specimens.

Methods: This experimental in vitro study was conducted at the College of Dentistry, Qassim University, Saudi Arabia, from September 2021 to January 2022. Sixty disc-shaped specimens of Cention N were prepared using custom-made moulds (10 mm diameter × 2 mm thickness). Specimens were randomly allocated into three groups (n = 20): Group 1 (control, Mylar strip finish), Group 2 (three-step Sof-Lex disc system), and Group 3 (one-step PoGo system). Half of each group was light-cured (40 seconds at 400 mW/cm²) while the remainder was self-cured. Surface roughness was measured using profilometry (Perthometer M1; Mahr GmbH, Göttingen, Germany) and microhardness assessed via Vickers hardness test (Vickers hardness number, or VHN). Data were analysed using two-way ANOVA and post hoc Bonferroni tests (p < 0.05).

Results: Significant differences in surface roughness existed between polishing systems (F = 156.731, p < 0.001), with the control group producing the smoothest surface (0.077 ± 0.055 μm), followed by PoGo (0.173 ± 0.039 μm) and Sof-Lex (0.238 ± 0.054 μm). All values remained below the 0.2 μm bacterial adhesion threshold. Significant interaction effects were found for both surface roughness and microhardness (p < 0.001). Light-cured specimens showed significantly higher microhardness than self-cured ones (47.8 ± 0.2 vs. 42.3 ± 0.2 VHN, p < 0.001).

Conclusion: The null hypothesis was rejected. While the Mylar strip finish provided optimal smoothness, all polishing systems maintained clinically acceptable surface roughness. Sof-Lex polishing combined with light curing offers superior microhardness, potentially enhancing restoration longevity.

## Introduction

Proper finishing and polishing of restorative materials are important steps to enhance both the aesthetics and longevity of restorations [1]. Increased plaque retention, gingival inflammation, irritation of the tongue, lips, and cheeks, and decreased gloss and increased discolouration of the material surface can result from rough surface texture [1]. Surface roughness values exceeding 0.2 μm have been shown to significantly increase bacterial adhesion and biofilm formation, establishing this as a critical threshold for clinical acceptability [2].

However, it is difficult to obtain a smooth surface on tooth-coloured materials at the end of polishing due to the shape, quantity, and size of the filler particles of the materials. Thus, the effect of polishing systems on surface roughness is material dependent due to their heterogeneous nature [1]. The recent literature has provided additional insights into these material-dependent responses [3,4]. Elnahas et al. [3] demonstrated that zirconium oxide-reinforced bulk-fill composites show varying responses to different polishing protocols, with multi-step systems achieving superior surface properties. Similarly, Kumari et al. [4] reported that alkaline glass fillers respond differently to mechanical polishing compared to traditional silica-based fillers, with roughness values ranging from 0.15 to 0.35 μm depending on the polishing protocol employed. These findings emphasize the necessity of material-specific polishing strategies for optimal clinical outcomes.

Recently, a new material family has been introduced to the market known as "alkasite". Cention N (Ivoclar Vivadent, Schaan, Liechtenstein) is a member of this family [5]. It is a urethane dimethacrylate (UDMA)-based, self-curing powder/liquid restorative with optional additional light curing [6]. The liquid is composed of dimethacrylates and initiators, while the powder contains various glass fillers, initiators, and pigments [6]. It is radio-opaque and contains alkaline glass fillers capable of releasing fluoride, calcium, and hydroxide ions [5-7]. Cention N shows low polymerisation shrinkage, reduced microleakage, and better marginal seal than other resin-based restorative materials [8]. Also, it exhibits comparable flexural strength to micro- and nano-hybrid resin composites [8-10]. It has excellent calcium and fluoride release, recharge capacity, and apatite formation ability compared to other bioactive materials and glass ionomer cement (GIC) [11,12].

While limited research has explored the surface properties of alkasite materials following different finishing protocols, a comprehensive evaluation of the combined effects of polishing systems and curing modes on both surface roughness and microhardness of alkasite restorative materials remains underexplored in the current literature [8]. The null hypothesis tested will be that there was no difference between three finishing, polishing protocols and curing mode in terms of microhardness and surface roughness.

## Materials and methods

Sample size was calculated using GPower 3.1.9.7 based on pooled data from previous studies [1,3-5]. Effect sizes were synthesized from reported surface roughness differences (0.08-0.35 μm) and microhardness variations (8-15 VHN, or Vickers hardness number), yielding a conservative Cohen's d of 1.5. With α = 0.05, power = 0.80, and six groups (3 polishing × 2 curing modes), GPower indicated a minimum of eight specimens per group. Including a 25% safety margin, 60 disc-shaped specimens (10 per subgroup) were prepared. This laboratory study was conducted following the Declaration of Helsinki principles, with ethical approval granted by the Qassim University IRB (EA/6067/2021).

Sixty disc-shaped specimens of Cention N were prepared using a custom-made mould with dimensions of 10 mm in diameter and 2 mm in thickness [6]. The material was mixed according to the manufacturer's instructions [6], placed into the mould, and then gently condensed to eliminate any voids. A transparent Mylar strip was applied to the surface, followed by a glass slab to produce a smooth and uniform finish.

The specimens were equally divided into self-cured and dual-cured groups. For the dual-curing process, the specimens were light-cured for 40 seconds using a curing unit (VALO; Ultradent Products, Inc., South Jordan, USA) with a light intensity of 400 mW/cm². The light-curing tip was positioned perpendicular and in close proximity to the specimen surface. After removing the samples from the mould, light curing was performed for an additional 20 seconds to ensure complete polymerisation. The specimens were then stored in distilled water at 37°C for 24 hours prior to polishing to simulate oral conditions.

After storage, the specimens were randomly allocated to three groups (n = 20) according to the polishing protocol. Group 1 served as the control group, with no further polishing beyond the Mylar strip finish. Group 2 was polished using a three-step Sof-Lex disc system (3M ESPE, St. Paul, USA) in the sequence of medium, fine, and extra fine discs. Group 3 was polished using a one-step PoGo system (Dentsply Caulk, Milford, USA). Manufacturers' instructions were followed during the polishing procedures. The discs were polished with light intermittent pressure for 20 seconds with a handpiece speed of 10,000 rpm. All polishing procedures were performed by a single trained examiner. Intra-examiner reliability was assessed by re-measuring 10% of specimens (n = 6) after 24 hours. The intraclass correlation coefficient (ICC) demonstrated excellent reliability for surface roughness (ICC = 0.94, 95% CI: 0.82-0.98) and microhardness (ICC = 0.92, 95% CI: 0.78-0.97).

**Figure 1:**
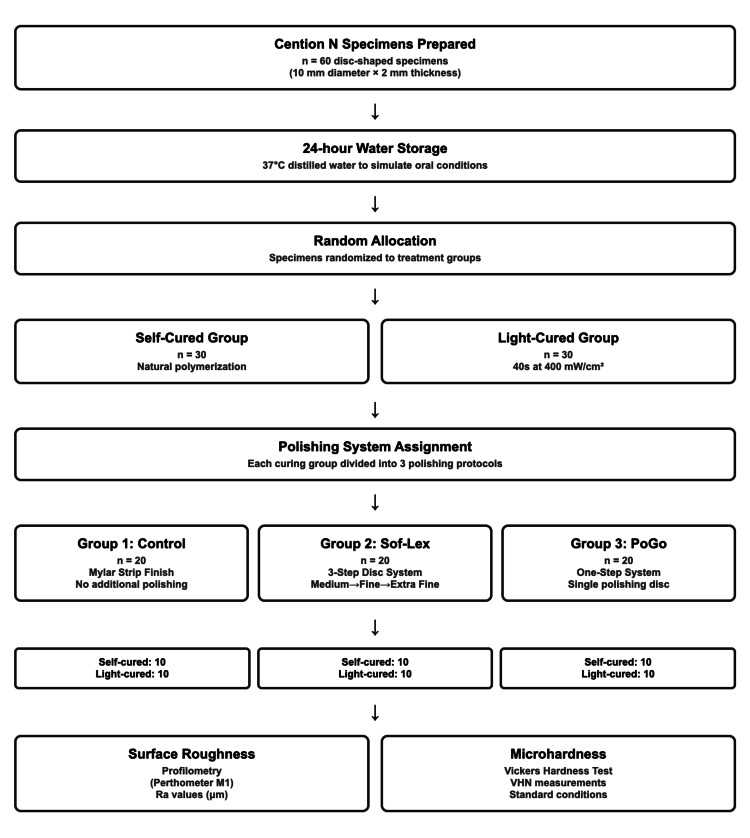
Study methodology flowchart showing specimen preparation, randomization, curing protocols, polishing procedures, and surface analysis measurements

Following polishing, each specimen was subjected to surface characterisation tests. Surface microhardness was evaluated using the Vickers hardness test. Surface roughness was measured using a profilometer (Perthometer M1; Mahr GmbH, Göttingen, Germany), following standardised measurement conditions for all samples (Figure 1).

Prior to statistical analysis, data distribution normality was assessed using the Shapiro-Wilk test due to the sample size (n < 50 per group). Homogeneity of variances was evaluated using Levene's test. Parametric two-way analysis of variance (ANOVA) was used to assess the influence of the polishing system on surface microhardness and roughness. Where statistically significant differences were found, post hoc Bonferroni tests were performed for pairwise comparisons. A p-value of less than 0.05 was considered statistically significant. All statistical analyses were performed using IBM SPSS Statistics, version 21 (IBM Corp., Armonk, NY).

## Results

A total of 60 specimens were used to complete the study protocol and were included in the statistical analysis. Shapiro-Wilk tests confirmed normal distribution for both surface roughness (p = 0.089-0.654) and microhardness (p = 0.112-0.743) data across all groups (p > 0.05). Levene's test confirmed homogeneity of variances for surface roughness (p = 0.156) and microhardness (p = 0.203). These results satisfied the requirements for parametric two-way ANOVA procedures.

Surface roughness results

Descriptive statistics for surface roughness measurements are presented in Table 1. The control group (Mylar strip finish) achieved the smoothest surface with a mean roughness of 0.077 ± 0.055 μm, followed by PoGo (0.173 ± 0.039 μm) and Sof-Lex (0.238 ± 0.054 μm). All individual group means remained well below the critical 0.2 μm threshold for bacterial adhesion.

**Table 1:** Surface roughness descriptive statistics (Ra, μm) Surface roughness (unit, Ra) is measured in micrometers (μm). All surface roughness values remain below the 0.2 μm bacterial adhesion threshold. Total sample size, n = 60 (10 per group combination).

Polishing system	Self-cured (chemical) mode			Light-cured mode			Combined mean ± SD (n = 20)
	Mean ± SD	95% CI	n	Mean ± SD	95% CI	n	
Control (Mylar)	0.130 ± 0.009	0.124-0.136	10	0.025 ± 0.016	0.014-0.036	10	0.077 ± 0.055
PoGo	0.160 ± 0.047	0.127-0.193	10	0.186 ± 0.025	0.168-0.204	10	0.173 ± 0.039
Sof-Lex	0.194 ± 0.036	0.169-0.219	10	0.283 ± 0.025	0.265-0.301	10	0.238 ± 0.054
Overall	0.161 ± 0.042	-	30	0.165 ± 0.110	-	30	0.163 ± 0.083

Two-way ANOVA results for surface roughness (Table 2) revealed a highly significant main effect for the polishing system (F = 156.731, p < 0.001, partial η² = 0.853), indicating substantial between-group differences. However, no significant effect was found for curing mode (F = 0.188, p = 0.667, partial η² = 0.003). There was no significant interaction between the polishing system and curing mode (F = 0.075, p = 0.928), indicating that the polishing systems worked similarly regardless of how the material was cured.

**Table 2:** Two-way ANOVA results for surface roughness (Ra, μm) All F-values represent between-group comparisons. All surface roughness values remain below the 0.2 μm bacterial adhesion threshold. Total sample size, n = 60 (10 per group combination). *Statistically significant at α = 0.05.

Source of variation	Sum of squares	df	Mean square	F-value	p-value	Partial η²	Significance
Polishing system	0.312	2	0.156	156.731	<0.001*	0.853	Significant
Curing mode	0.000	1	0.000	0.185	0.667	0.003	Not significant
Polishing × curing interaction	0.001	2	0.0005	0.075	0.928	0.003	Not significant
Error (within-groups)	0.004	54	0.00007	-	-	-	-

Post hoc Bonferroni comparisons (Table 3) confirmed that all pairwise differences between polishing systems were statistically significant (all p < 0.001). The mean differences were as follows: control vs. PoGo, -0.096 μm, 95% CI: -0.118 to -0.073; control vs. Sof-Lex, -0.161 μm, 95% CI: -0.183 to -0.138; and PoGo vs. Sof-Lex, -0.065 μm, 95% CI: -0.088 to -0.042. All comparisons demonstrated large effect sizes (Cohen's d > 0.8), confirming the clinical significance of the differences. No post hoc analysis was required for curing modes due to the non-significant ANOVA result.

**Table 3:** Surface roughness - post hoc multiple comparisons I = first comparison group; J = second comparison group. Negative values indicate that the first group (I) had lower values than the second group (J). *All pairwise comparisons between polishing systems are statistically significant (p < 0.001) with large effect sizes. No post hoc analysis was performed for curing modes due to the non-significant ANOVA effect. Cohen's d interpretation: small (0.2), medium (0.5), large (0.8).

Factor	Comparison (I vs. J)	Mean difference (I - J) μm	Standard error	p-value	95% Confidence interval	Cohen's d (effect size)
Polishing system (between-groups)	Control vs. PoGo	-0.096	0.009	<0.001*	-0.118 to -0.073	2.8 (large)
	Control vs. Sof-Lex	-0.161	0.009	<0.001*	-0.183 to -0.138	3.5 (large)
	PoGo vs. Sof-Lex	-0.065	0.009	<0.001*	-0.088 to -0.042	1.9 (large)
Curing mode (between-groups)	Self-cured vs. light-cured	-0.004	0.008	0.667	-0.021 to +0.013	0.05 (none)

Microhardness results

Descriptive statistics for microhardness measurements are presented in Table 4. The Sof-Lex polishing system demonstrated the highest microhardness values (59.0 ± 0.3 VHN), followed by PoGo (40.6 ± 0.3 VHN) and the control group (35.6 ± 0.3 VHN). Light-cured specimens consistently exhibited superior microhardness compared to self-cured specimens across all polishing systems, with overall means of 47.8 ± 0.2 VHN and 42.3 ± 0.2 VHN, respectively.

**Table 4:** Microhardness descriptive statistics (VHN) VHN: Vickers hardness number. Total sample size, n = 60 (10 per group combination).

Polishing system	Self-cured (chemical) mode			Light-cured mode			Combined mean ± SE (n = 20)
	Mean ± SE	95% CI	n	Mean ± SE	95% CI	n	
Control	31.2 ± 0.4	30.5-31.9	10	39.9 ± 0.4	39.2-40.6	10	35.6 ± 0.3
PoGo	38.8 ± 0.4	38.1-39.5	10	42.3 ± 0.4	41.6-43.0	10	40.6 ± 0.3
Sof-Lex	57.0 ± 0.4	56.3-57.7	10	61.1 ± 0.4	60.4-61.8	10	59.0 ± 0.3
Overall	42.3 ± 0.2	41.9-42.7	30	47.8 ± 0.2	47.4-48.2	30	45.1 ± 0.2

Two-way ANOVA (Table 5) revealed significant main effects for both polishing system (F = 2336.148, p < 0.001, partial η² = 0.989) and curing mode (F = 339.178, p < 0.001, partial η² = 0.863). A significant interaction effect was observed between polishing system and curing mode (F = 30.872, p < 0.001, partial η² = 0.533), indicating that the magnitude of light curing benefit varied depending on the polishing system employed.

**Table 5:** Two-way ANOVA results for microhardness (VHN) VHN: Vickers hardness number. *All main effects and interactions are statistically significant at α = 0.05. Large effect sizes indicate clinically meaningful differences between groups. Significant interaction requires simple effects analysis (see Table 6).

Source of variation	Sum of squares	df	Mean square	F-value	p-value	Partial η²	Significance
Polishing system	6116.512	2	3058.256	156.731	<0.001*	0.853	Significant
Curing mode	444.018	1	444.018	339.178	<0.001*	0.863	Significant
Polishing × curing interaction	80.828	2	40.414	30.872	<0.001*	0.533	Significant
Error (within-groups)	70.692	54	1.309	-	-	-	-

Post hoc Bonferroni comparisons (Table 6) revealed that all pairwise differences between polishing systems were statistically significant (all p < 0.001). The Sof-Lex system produced significantly higher microhardness than both PoGo (mean difference = -18.469 VHN, 95% CI: -19.363 to -17.575) and control groups (mean difference = -23.479 VHN, 95% CI: -24.373 to -22.585). PoGo demonstrated significantly higher microhardness than the control group (mean difference = -5.010 VHN, 95% CI: -5.903 to -4.116). All comparisons exhibited large effect sizes (Cohen's d > 0.8), confirming the clinical significance of these differences. Light-cured specimens showed significantly higher microhardness than self-cured specimens (mean difference = -5.441 VHN, 95% CI: -6.033 to -4.848, p < 0.001).

**Table 6:** Microhardness - post hoc comparisons and interaction analysis I = first comparison group; J = second comparison group. Negative values indicate that the first group (I) had lower values than the second group (J). *All comparisons significant at p < 0.001 with large effect sizes. The significant interaction (F = 30.872, p < 0.001) indicates that light curing benefits vary by polishing system.

Factor	Comparison (I vs J)	Mean difference (I - J) VHN	Standard error	p-value	95% Confidence interval	Cohen's d (effect size)
Polishing system (between-groups)	Control vs. PoGo	-5.010	0.362	<0.001*	-5.903, -4.116	13.8 (large)
	Control vs. Sof-Lex	-23.479	0.362	<0.001*	-24.373, -22.585	64.9 (large)
	PoGo vs. Sof-Lex	-18.469	0.362	<0.001*	-19.363, -17.575	51.0 (large)
Curing mode (between-groups)	Self-cured vs. light-cured	-5.441	0.295	<0.001*	-6.033, -4.848	18.4 (large)

## Discussion

The present study investigated the impact of curing mode and different polishing protocols on the surface roughness and microhardness of Cention N, a novel alkasite restorative material. The findings revealed significant differences between polishing systems, with important implications for clinical practice and restoration longevity.

The selection of surface roughness and microhardness as evaluation parameters in this study was based on their direct clinical relevance. Surface roughness is a critical determinant of bacterial adhesion, biofilm formation, and subsequent secondary caries development, while microhardness correlates with wear resistance and long-term durability of restorations [13]. These parameters together provide a comprehensive assessment of restoration quality and potential longevity, making them essential indicators for evaluating the clinical performance of novel restorative materials.

The study demonstrated statistically significant differences in surface roughness (Ra) between polishing systems (p < 0.001), with the control group (Mylar strip finish) producing the smoothest surface. This finding aligns with previous research by Erdemir et al. [1] as well as studies by Kaizer et al. [14] and Silva et al. [15] who reported that Mylar strip finishes typically provide superior smoothness compared to mechanical polishing systems due to the absence of abrasive particle interaction with the restoration surface. However, clinical situations often require contouring and adjustment, making post-polymerisation polishing inevitable [1]. Importantly, all surface roughness values in this study remained below the critical threshold of 0.2 μm for bacterial adhesion, suggesting that regardless of the polishing system employed, the alkasite material maintained clinically acceptable surface characteristics [16].

The relatively low surface roughness values achieved with all polishing systems may be attributed to the unique filler composition of Cention N. Unlike conventional resin composites, alkasite materials contain alkaline glass fillers with different morphological characteristics that may respond more favourably to mechanical polishing procedures [5,6]. A recent study emphasised the importance of material-specific polishing protocols, particularly for newer bioactive materials like alkasites that are gaining popularity in dental practices [17].

The Sof-Lex polishing system demonstrated significantly higher microhardness values compared to other groups (p < 0.001). This finding can be explained by the progressive grit sequence of the Sof-Lex system (medium to extra-fine) that may have contributed to surface densification through controlled abrasion, resulting in a more compact surface layer [7]. The study revealed significant interaction effects for both surface roughness and microhardness, indicating that curing mode effects vary depending on the polishing system employed. Light-cured specimens demonstrated significantly higher microhardness compared to self-cured samples (p < 0.001), attributed to an enhanced degree of conversion achieved through photopolymerisation [7].

A key strength of the present study compared to the previous research is the simultaneous evaluation of both polishing system effects and curing mode interactions in relation to alkasite materials. While previous studies have examined either surface roughness or microhardness in isolation [18,19], our comprehensive approach provides a more complete understanding of how these variables interact to influence restoration quality. Additionally, our study is among the first to systematically compare one-step versus multi-step polishing protocols specifically for alkasite materials, addressing a gap in the existing literature.

The findings have important clinical implications for dental practice. The observation that all polishing systems maintained surface roughness below the critical threshold for bacterial adhesion suggests that clinicians have flexibility in choosing polishing protocols based on convenience and efficiency. However, the superior microhardness achieved with the Sof-Lex system suggests potential advantages of restoration longevity, particularly in high-stress areas [20]. The finding that light curing enhances microhardness supports the recommendation for dual-curing protocols when possible, which is especially relevant for alkasite materials used in stress-bearing areas due to their bioactive properties [21]. Recent clinical studies have demonstrated excellent performance of alkasite materials in paediatric populations [21]. Contemporary research on bulk-fill composites also supports the effectiveness of multi-step polishing protocols for achieving optimal surface properties [3].

Cention N's unique composition as an alkasite material presents distinct advantages through fluoride, calcium, and hydroxide ion release, positioning these materials as valuable additions to the restorative armamentarium, particularly in regions where preventive dental care may be limited [11].

Several limitations should be acknowledged when interpreting these findings. First, this in vitro investigation was conducted under controlled laboratory conditions that may not fully replicate the complex oral environment, including factors such as saliva, thermal cycling, masticatory forces, and oral biofilms, which limits the direct extrapolation of our study findings to clinical scenarios. Second, the study evaluated only one alkasite material (Cention N) with immediate post-polishing assessment, and findings may not be generalisable to other alkasite formulations or reflect long-term surface property changes under clinical conditions. Third, while this study focused on surface roughness and microhardness, other important clinical parameters, such as wear resistance, colour stability, and long-term bioactivity maintenance, were not assessed, which are crucial for comprehensive material evaluation. Future research should include clinical studies to validate these laboratory findings and evaluate long-term performance under actual oral conditions.

## Conclusions

Based on the findings of this in vitro study, the null hypothesis was rejected as significant differences were observed between polishing systems in terms of both surface roughness and microhardness of the Cention N alkasite restorative material. The Mylar strip finish produced the smoothest surface, while the Sof-Lex system achieved the highest microhardness values. All polishing systems maintained surface roughness below the 0.2 μm bacterial adhesion threshold. Light-cured specimens consistently demonstrated higher microhardness than self-cured specimens across all systems tested. These laboratory findings demonstrate that different polishing protocols produce varying surface characteristics in alkasite materials. Further clinical studies are needed to validate these findings under oral conditions.
